# About a Large Botryoid Rhabdomyosarcoma in a Little Girl: Management Difficulties and Literature Review

**DOI:** 10.1155/2023/4789851

**Published:** 2023-01-27

**Authors:** Dehi Boston Mian, Vedi Andre Serges Loue, Sylvanus Koui

**Affiliations:** ^1^Department of Gynecology and Obstetrics, University Hospital of Cocody, Abidjan, Côte d'Ivoire; ^2^Faculty of Medicine, Felix Houphouët Boigny University of Cocody, Abidjan 01 BP V13, Côte d'Ivoire; ^3^Department of Anatomopathology-Pathology of the University Hospital of Cocody, Abidjan, Côte d'Ivoire

## Abstract

**Background:**

Rhabdomyosarcoma (RMS) is a rare high-grade malignant tumor and the most common soft-tissue sarcoma, which occurs in young girl over 5 years old. Multimodality treatment associating with surgery, chemotherapy, and/or radiotherapy culminate in a >70% overall 5-year survival. This is the first case reported in 30 years of practice in Côte d'Ivoire, low- and middle-income country (LMIC).

**Objective:**

To summarize clinical data, the significant alternative chemotherapy efficiency and difficulties related to the prognosis evaluation in an LMIC.

**Case:**

A 2-year-old girl had been examined for a large mass in the vulvar region and clitoris. We carried out a biopsy for histopathologist exam. This allows pathologic, genetic, and biological characterization of nonmetastatic botryoid rhabdomyosarcoma. A multidisciplinary team decision of neoadjuvant chemotherapy was retained combining vincristine, cyclophosphamide, and actinomycin D or alternatively with Adriamycin. After 3 weeks of chemotherapy, significant volumetric reduction of tumor was observed. Yet a surgical removal was proposed but not performed because the patient has no longer consulted our medical center and was lost to follow-up. Therefore, we cannot assess the long-term evolution and prognosis.

**Conclusion:**

Embryonal RMS (ERMS) of clitoris is a rare malignant tumor of infant. Histology and immunohistochemistry are essential for diagnostic but unavailable in our context. We want to emphasize on the difficulties encountered in treatment and prognosis assessment. The primary free surgical removal of the vulva with adjuvant chemotherapy and/or radiotherapy must then be implemented in our practice.

## 1. Introduction

Rhabdomyosarcoma (RMS) is a high-grade soft-tissue malignant sarcoma, commonly found in children and adolescents compared to adults [1, 2]. RMS represented 50% of all malignant tumor of vulva in pediatric patients (≤21 years) for the 50-year period between 1970 and 2020. [2]. This malignant tumor can be located anywhere in the human body. The predilection primary site among children and adolescents is the genitourinary tract (20–25%), infrequently in the vulva and clitoris region [3]. Histologically, RMS are high-grade neoplasms originating from primitive mesenchymal tissue. Approximately 90% of cases are embryonal histology [4]. The clitoral location was very severe due to highly invasive character and early death. Over the last 30 years, patient outcomes have improved with the incorporation of multimodal therapies, including chemotherapy, radiation therapy, and surgery [5]. An earlier treatment could sometimes ensure better prognosis, but the evolution was usually severe. Improved overall survival (OS) rates have led to a renewed emphasis on reducing local morbidity and chemotherapy toxicity [3, 5]. We report the first embryonal RMS (eRMS) of the clitoris in a child, and focus on the results of the most recent clinical trials to discuss the efficiency of an alternative chemotherapy as advancement in treatment in low- and middle-income countries (LMICs) and the difficulties of long-term medical follow-up.

## 2. Case Presentation

**Figure 1:**
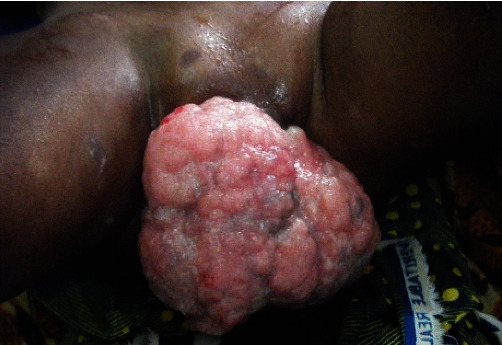
Significant (12 cm × 15 cm) tumoral spread to the whole vulvar and clitoris region.

**Figure 2:**
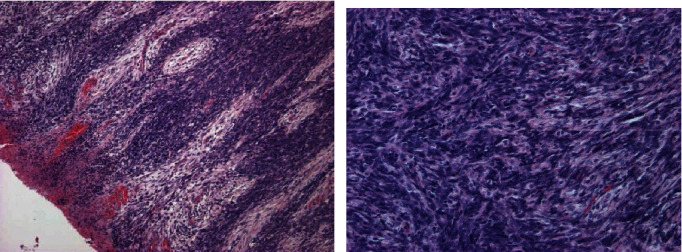
Histological characteristics of the vulval tumor. (a) Abnormal proliferation of spindle and ovoid cells (hematein-eosin ×100). (b) Tumoral proliferation of basophilic cytoplasm cells, with elongated or oval nuclei and anisocaryosis (hematein-eosin ×400).

**Figure 3:**
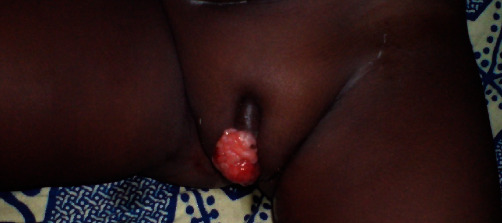
Macroscopical view of clitoris features after chemotherapy cycles.

A 2-year-old girl was admitted to the Gynecology Department of teaching hospital of Cocody in Côte d'Ivoire (West Africa). She presented a quickly spreading large vulval swelling during the past 3 months. The volume of the mass had gradually increased from a pigeon egg size to a pineapple size. Gynecological examination revealed a crumbly cauliflower-like firm tumor of vulvar region and clitoris with bleeding areas. The swelling size was approximately measuring 12 cm × 8 cm (Figure 1). No inguinal adenopathy was found, and the other organs were unremarkable. No ultrasonic examination, CT, or MRI scan was done because of family's limited resources. A biopsy was performed for a histologic analysis with the sampling fixed with 10% formaldehyde. Histological examination revealed combined areas of abnormal proliferation layers of spindle and ovoid cells (Figure 2(a)). These abnormal cells also had a basophilic cytoplasm and elongated or oval anisokaryosis nuclei (Figure 2(b)). The immunohistochemistry analysis was done at ERASME Pathology Laboratory of Bruxelles (Belgium). It showed genetic features like anti-myogenic and anti-actin antibodies (Figure 2(b)). Diagnosis of eRMS was then confirmed by both histological and immunohistochemistry data. No tumoral extension or metastatic location was found in a pelvic CT scan performed for extension research. A multidisciplinary team decision (MTD) then suggested a neoadjuvant chemotherapy before tumor surgical removal. Two ambulatory chemotherapy regimens of three drugs were alternatively conducted every week during a period of 3 weeks. The first one (VAC) associated Vincristine (1.5 mg/m^2^ IV) and Actinomycin D (15 mcg/kg IV) on day 1, with Cyclophosphamide (20 mg/m^2^/day IV) from day 1 to day 5. The second chemotherapy combination (VAD) substitutes Actinomycin D with Adriamycin (60 mg/m^2^ IV). She received no X-ray therapy, as unavailable in our country at this time. After 3 weeks of chemotherapy, we observed a significant reduction in the tumoral size, close to 75% (Figure 3). A surgical removal of the remaining mass was suggested. But the family had no health insurance and could no longer pay for the medical expenses. We then decided to carry on with chemotherapy since it was free. The outcome was a continuous decrease of the vulval tumor size for the next 3 months. Hence, an 80% tumoral decrease was achieved in 6-month treatment. The girl and her family have not come for consultation since this last chemotherapy treatment, so that long-term prognosis cannot be properly assessed because patient was lost to follow-up.

## 3. Discussion

Primary RMS of vulva is exceptional in young girls. It is a high-grade malignant neoplasm. Over the last 30 years, patient outcomes have improved with the incorporation of multimodal therapies, including chemotherapy, radiotherapy, and surgery [1, 5]. A bi-modal distribution of RMS was described with peaks between ages 2–4 and 12–16. Nearly 80% of RMS are diagnosed by the age of 14 [6]. The disease begins with no specific symptoms like a cluster-like vulval swelling. The diagnosis confirmation was made by histologic examination [7]. Histologically, tumors are high-grade neoplasms originating from primitive mesenchymal tissue and resembling skeletal muscle histogenesis [4]. The World Health Organization Classification of Soft Tissue and Bone tumors identifies prognostically different subtypes: eRMS, alveolar RMS (aRMS), pleomorphic, and spindle cell/sclerosing [4]. The two most common subtypes are eRMS (38.8%) and aRMS (22.3%) [3–8]. Immunohistochemistry is essential to avoid diagnostic errors [3–8]. Most cases of genitourinary RMS occur sporadically, although associated with several genetic syndromes, such as the Li-Fraumeni syndrome (18–27%), which is caused by the loss-of-function germline mutations in TP53 [9], the DICER1 syndrome [10], and several other cancer predisposition syndromes like Beckwith-Wiedemann syndrome, neurofibromatosis type 1, Gorlin's basal cell nevus, and Rubinstein-Taybi syndrome [11, 12]. None of these genetic predisposal factors could be researched in this case because no specialized genetic research laboratory was implemented in our country. No reports have described the typical clinical symptoms and features of RMS, so the histopathological findings are regarded as the diagnosis gold standard [13]. Eradication of a major part of primary malignant clitoris tumor requires the use of combination of many procedures such as surgery and/or radiotherapy, and systemic cures of chemotherapy [1, 2, 14, 15]. Systemic chemotherapy using alkylator-containing regimen is the standard first-line option. It is an intensive treatment combining vincristine and actinomycin D. The alkylator used in North America is cyclophosphamide (VAC combination), whereas ifosfamide (IVA combination) is typically used in Europe [14, 15, 19]. After a multidisciplinary team meeting, we decided to use the VAC regimen and then substituted Actinomycin D with Adriamycin in the second week due to unavailability. In this case, we did not find local or general metastases; however, all patients with RMS were presumed to have a micrometastatic disease whose eradication requires the achievement of both local and systemic treatment. Chemotherapy allowed systemic control even in localized forms of the disease [16]. The main objective of the treatment was local tumor reduction and metastatic occurrence prevention. The chemotherapy regimens, generally included a combination of effective agents, such as cyclophosphamide, actinomycin D, cisplatin, carboplatin, vincristine, and etoposide [10, 11, 14, 16, 17]. Our case showed a significant efficiency of this chemotherapy regimen with significant decrease of the tumor size after only 3 weeks of treatment (Figure 3). However, regarding treatment and prognosis, we think that our patient could receive most exhaustive treatment. Indeed, this young girl and her family did not return back to consultation for surgical removal of vulva. We could not make an objective assessment of the OS time even though the significant tumoral decrease was an encouraging result. For many authors in literature, tumoral prognosis is severe [18–20]. Some authors suggested that most patients are stratified into high-, intermediate-, and low-risk groups based on tumor size, tumor invasiveness, nodal status, primary tumor site, and pathological and molecular parameters (PAX-fusion status) [18, 19]. In other words, prognosis for RMS depends on primary tumor site, age, completeness of resection, presence and number of metastatic sites, histology, and biology of the tumor cells [18–20]. In addition, the overall cure rates have improved significantly thanks to the multimodal therapies, including surgery, radiotherapy, and chemotherapy (with various combinations of vincristine, actinomycin D, cyclophosphamide, etoposide, irinotecan, or ifosfamide). In recent studies, it was found that surgical therapy is the key basis and significant treatment for a better prognosis in patients [20]. The mean survival time with patients who underwent surgery is 16.7 months, whereas patients with non-surgical therapy is only of 9.5 months. It's important to report that our patient has been lost to follow-up. The parents' phone line was disconnected, and it is therefore really difficult to assess the prognosis. In addition, concomitant radiotherapy that allows prognosis improvement [3, 4] was unavailable in our country at the time of management. This young girl had an incomplete treatment as living in low socio-economic conditions with no health insurance or subsidies. The malignant tumor surgical removal had not been performed as the family never came back after chemotherapy treatment. Perhaps, the quick decrease of the tumor size made the parents believe that the girl was cured, or we were just not informed of her death. We could only suppose that because our efforts to reach them by phone were unsuccessful. In short term, we can say that the prognosis was good based on the speed tumoral decreased (Figure 3). In literature, some unfavorable factors have been identified such as age (>10 years), alveolar histological subtype, size (>5 cm), extragenital location of primary tumor, as well as prior existence of extensive regional nodal tumor sites or metastases [21]. Only the big tumoral size has been described here. This risk factor allows a classification to optimize and improve the treatment [19]. The patients with metastatic locations still have a poor prognosis in OS time (0–30%). Indeed, patients in the low-risk group have the best prognosis, namely recurrence-free survival (3-year RFS rate 88%) and OS [18]. The occurrence of recurrences is common in our LMICs due to long delays in care and discontinuation of treatment [15, 21].

## 4. Conclusion

RMS of clitoris is a rare malignant tumor. We want to emphasize on the incomplete treatment despite the efficiency of the alternative chemotherapy. The prognosis assessment was difficult because the patient was lost to follow-up, so the primary free surgical removal of the vulva with adjuvant chemotherapy and/or radiotherapy must be implemented in our practice.

## Data Availability

To access the data supporting the conclusions of the study, join Mian Dehi Boston to bostondehimian@yahoo.fr.
